# Combined Cardiac Risk Factors Predict COVID-19 Related Mortality and the Need for Mechanical Ventilation in Coptic Clergy

**DOI:** 10.3390/jcm10102066

**Published:** 2021-05-12

**Authors:** Michael Y. Henein, Ibadete Bytyçi, Rachel Nicoll, Rafik Shenouda, Sherif Ayad, Matteo Cameli, Federico Vancheri

**Affiliations:** 1 Institute of Public Health and Clinical Medicine, Umea University, 90187 Umea, Sweden; i.bytyci@hotmail.com (I.B.); rachelnicoll25@gmail.com (R.N.); rafik.sheneuda@umu.se (R.S.); 2 Molecular and Clinic Research Institute, St George University, London SW17 0QT, UK; 3 Institute of Fluid Dynamics, Brunel University, London UB8 3PH, UK; 4 International Cardiac Centre, Alexandria 21526, Egypt; 5 Department of Cardiology, Faculty of Medicine, Alexandria University, Alexandria 21526, Egypt; sherifwagdyayad@yahoo.com; 6 Department of Cardiovascular Disease, University of Siena, 53100 Siena, Italy; matteo.cameli@yahoo.com; 7 Department of Internal Medicine, S. Elia Hospital, 93100 Caltanissetta, Italy; federico.vancheri@ki.se

**Keywords:** COVID-19, Coptic clergy, mortality, cardiovascular risk factors

## Abstract

Background and Aims: The clinical adverse events of COVID-19 among clergy worldwide have been found to be higher than among ordinary communities, probably because of the nature of their work. The aim of this study was to assess the impact of cardiac risk factors on COVID-19-related mortality and the need for mechanical ventilation in Coptic clergy. Methods: Of 1570 Coptic clergy participating in the COVID-19-Clergy study, serving in Egypt, USA and Europe, 213 had the infection and were included in this analysis. Based on the presence of systemic arterial hypertension (AH), participants were divided into two groups: Group-I, clergy with AH (*n* = 77) and Group-II, without AH (*n* = 136). Participants’ demographic indices, cardiovascular risk factors, COVID-19 management details and related mortality were assessed. Results: Clergy with AH were older (*p* < 0.001), more obese (*p* = 0.04), had frequent type 2 diabetes (DM) (*p* = 0.001), dyslipidemia (*p* = 0.001) and coronary heart disease (CHD) (*p* = 0.04) compared to those without AH. COVID-19 treatment at home, hospital or in intensive care did not differ between the patient groups (*p* > 0.05 for all). Clergy serving in Northern and Southern Egypt had a higher mortality rate compared to those from Europe and the USA combined (5.22%, 6.38%, 0%; *p* = 0.001). The impact of AH on mortality was significant only in Southern Egypt (10% vs. 3.7%; *p* = 0.01) but not in Northern Egypt (4.88% vs. 5.81%; *p* = 0.43). In multivariate analysis, CHD OR 1.607 ((0.982 to 3.051); *p* = 0.02) and obesity, OR 3.403 ((1.902 to 4.694); *p* = 0.04) predicted COVID-19 related mortality. A model combining cardiac risk factors (systolic blood pressure (SBP) ≥ 160 mmHg, DM, obesity and history of CHD) was the most powerful independent predictor of COVID-19-related mortality, OR 3.991 ((1.919 to 6.844); *p* = 0.002). Almost the same model also proved the best independent multivariate predictor of mechanical ventilation OR 1.501 ((0.809 to 6.108); *p* = 0.001). Conclusion: In Coptic clergy, the cumulative impact of risk factors was the most powerful predictor of mortality and the need for mechanical ventilation.

## 1. Introduction

COVID-19 is an aggressive pandemic that has claimed the lives of millions worldwide [1], and many of those recovering may develop serious long-term symptoms. First wave studies [2,3] reported a higher mortality rate among the black, Asian and minority ethnic (BAME) communities, highlighting the importance of ethnic impact on the natural history of the disease. In addition, most sufferers requiring mechanical ventilation have been found to have significant co-morbidities [4]. On the other hand, social distancing has played an important role in controlling, to a great extent, the rate of disease transmission with its associated mortality [5,6], and vaccination has provided significant protection, particularly among the elderly [7,8].

We have previously reported high COVID-19 prevalence among Coptic clergy and explained it on the basis of their lifestyle and regular community service, which requires close contact with their parishioners. We have also highlighted the important role of obesity in explaining this high disease prevalence [9]. The aim of this study is to assess the additional role of conventional cardiovascular risk factors in predicting mortality and the need for mechanical ventilation in Coptic clergy with COVID-19.

## 2. Methods

### 2.1. Study Design and Patients

**Figure 1:**
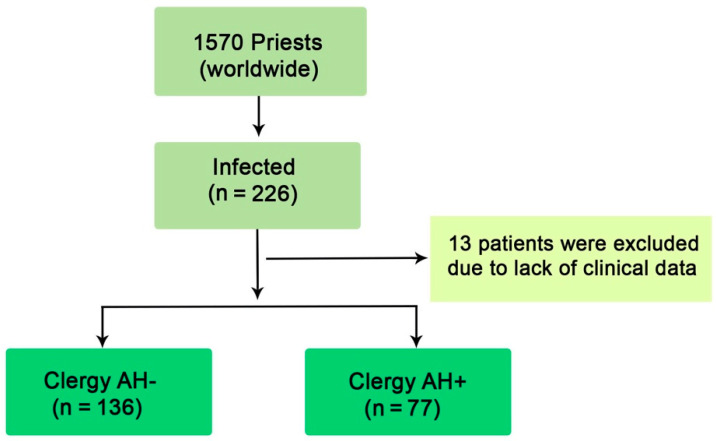
Flow chart of participants.

The present study is a retrospective evaluation of a cohort of 1576 Coptic clergy worldwide, from March to December 2020. It is a sub-study within the COVID-19-CVD international study, which is investigating the impact of COVID-19 on the cardiovascular system and which has been approved by the Swedish Ethics Board (Dnr 2020-02217 Stockholm avdelning 2 medicin). M.Y.H. (The principal investigator) designed the study protocol which was endorsed by the Head of the Coptic Church in Egypt. 1570 clergy within 25 dioceses were evaluated from different areas of Egypt, Europe and the USA. Dioceses in Egypt were divided into two main regions, Northern (comprising Alexandria, the Delta and Cairo) and Southern (comprising all cities geographically south of Cairo). Data collected from Coptic clergy serving in the European countries and the USA were combined and analyzed as one group since they mostly follow similar disease prevention and treatment strategies. According to the presence of arterial hypertension (AH), the clergy suffering COVID-19 were divided into two groups: Group-I: clergy with AH (*n* = 77) and Group-II: clergy without AH (*n* = 136). 13 infected clergy were excluded due to lack of clinical data (Figure 1).

### 2.2. Cardiovascular Risk Factor Assessment

Cardiovascular risk factors such as arterial hypertension (AH), type 2 diabetes mellitus (DM), coronary heart disease (CHD), dyslipidemia, obesity and family history of cardiovascular disease were assessed based on medical records and prior investigations and management. According to conventional international risk factor assessment and cut-off values for body mass index (BMI), overweight was defined as BMI of 25–29.9 kg/m^2^ and obesity as BMI ≥30 kg/m^2^. Systemic AH was diagnosed when systolic blood pressure (SBP) was ≥130 mmHg and/or diastolic blood pressure (DBP) was ≥80 mmHg. Type 2 diabetes mellitus (DM) was identified based on pre-recruitment diagnosis leading to participants commenced on conventional oral hypoglycemics and/or insulin therapy. Dyslipidemia was determined from medical records or if the individual had been commenced on statins. Evidence for coronary artery disease was also evaluated from medical records, based on prior investigations and management.

### 2.3. Clinical Events

Clinical events (CE) were retrospectively collected and information on participants’ clinical outcome was obtained from electronic medical records, clinical visits, personal communication with general physicians and confidential telephone interviews with patients and relatives. The study’s primary outcome was COVID-19-related mortality; the secondary outcome was the need for mechanical ventilation.

### 2.4. Statistical Analysis

Discrete data are reported as frequencies (percentages) and continuous variables are shown as means and standard deviation (SD) if normally distributed, or median and interquartile range (IQR: Q1–Q3) in case of skewed distribution. Continuous data were compared with the two-tailed Student *t*-test and discrete data with Chi-square test. Analysis of variance and Bonferroni statistical tests were used to compare quantitative variables between more than two groups. Predictors of mortality related to COVID-19 and the need for mechanical ventilation were identified with univariate analysis. Independent predictors were identified using multivariate logistic regression analysis using the stepwise method. A significant difference was defined as *p*-value < 0.05 (two-tailed). Statistical analysis was performed with SPSS Software Package version 26.0 (IBM Corp., Armonk, NY, USA).

## 3. Results

### 3.1. Demographic and Clinical Indices for Clergy with COVID-19

Two hundred and thirteen symptomatic clergy with COVID-19 were included in the study. Patients’ mean age was 49.6 ± 12 years and all were males. Out of the 213 clergy, 122 (57.3%) were obese, 59 (27.7%) had diabetes, 68 (31.9%) had dyslipidemia and 20 (9.4%) had coronary artery disease. 171/213 (80.2%) of the clergy were treated at home and the remaining 26 (16.9%) in hospital, 17 (7.9%) of them needed intensive care management and 15 (7.1%) required mechanical ventilation (Table 1 and Table 2).

### 3.2. Demographic and Clinical Data of Clergy with and without AH

Compared to clergy without AH, the prevalence of COVID-19 was higher in those with AH (20.1% vs. 10.2%). Clergy with AH were older (*p* < 0.001), more obese (*p* = 0.04), had more diabetes (*p* = 0.001), dyslipidemia (*p* = 0.001) and coronary heart disease (CHD) (*p* = 0.04) compared to those without AH. Family history for cardiovascular disease, stroke or CHD did not differ between patient groups (*p* > 0.05 for both). The frequency of management at home, hospital or intensive care did not differ between patient groups with or without AH (*p* > 0.05 for all, Table 1).

### 3.3. Geographical Impact on Clinical Events

Among the 213 clergy with COVID-19, the overall mortality rate was 4.69% and the need for mechanical ventilation was 7.1% (Table 2). Based on geographical analysis, the overall clergy mortality rate in Northern (*n* = 136, 51 with AH) and Southern Egypt (*n* = 46; 14 with AH) was higher compared to that in Europe and USA combined (5.22%, 6.51%, 0%, respectively; *p* = 0.001). The impact of AH on mortality was not significant in Northern (5.88% vs. 4.71%, respectively; *p* = 0.22) and Southern Egypt (7.1% vs. 6.21%, respectively; *p* = 0.43) compared to those without AH (Table 3).

### 3.4. Distribution of Cardiac Risk Factors among Clergy with and without Adverse Clinical Events

**Figure 2:**
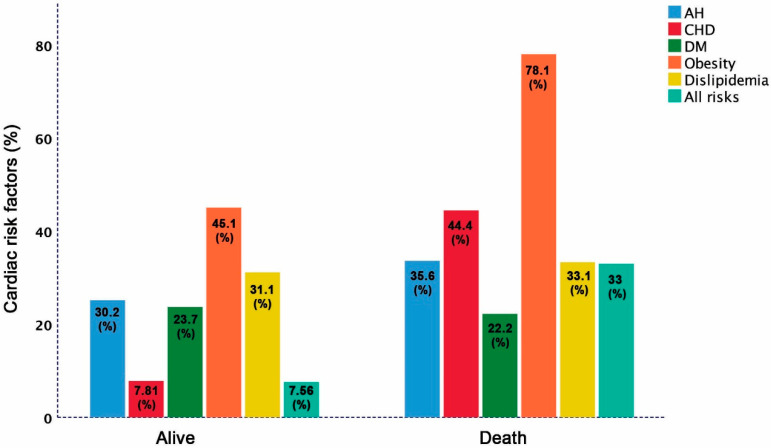
Distribution of cardiac risk factors among living and deceased clergy.

**Figure 3:**
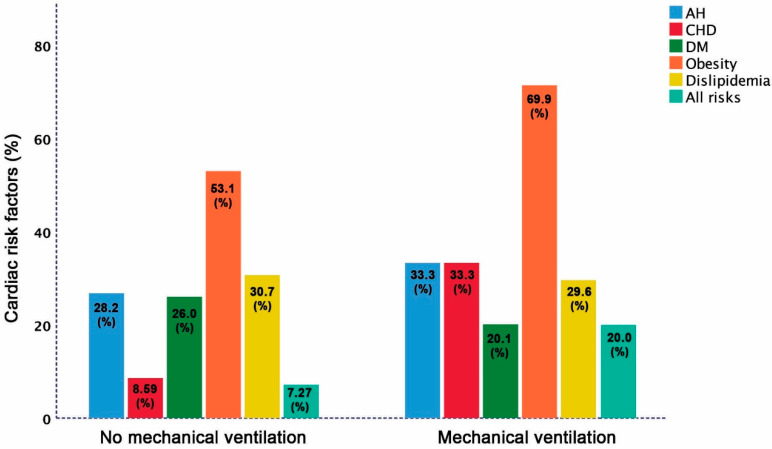
Distribution of cardiac risk factors among clergy based on the need for mechanical ventilation.

The overall mortality rate in the study participants was 4.69% with no difference between clergy with and without AH (4.41% vs. 5.19% *p* = 0.12). The prevalence of risk factors among the deceased clergy compared to survivors were: CHD (44.4% vs. 7.81%; *p* = 0.001), obesity (78.1 vs. 45.1%, *p* < 0.001), and AH trends to be more prevalent (35.6% vs. 30.2%; *p* = 0.052), but DM (22.2% vs. 23.7%; *p* = 0.44), and dyslipidemia (33.1% vs. 31.1%; *p* = 0.18) were not different. However, the combined cardiac risk factors (AH, CHD, DM, obesity and dyslipidemia) were more prevalent among deceased clergy compared to survivors (33% vs. 7.56%; *p* < 0.001 Figure 2). Likewise, combined risk factors were more prevalent in the 15 (7.1%) clergy requiring mechanical ventilation compared to those who did not (20% vs. 7.27%; *p* < 0.001) (Figure 3).

### 3.5. Predictors of COVID-19-Related Adverse Clinical Events

Mortality prediction: In univariate analysis, DM (*p* = 0.02), obesity (*p* = 0.03), AH (0.04) and CHD (*p* = 0.001) predicted COVID-19-related mortality. In multivariate analysis, only CHD (OR 1.607 ((0.982 to 3.051); *p* = 0.02)) and obesity (OR 3.403 ((1.902 to 4.694; *p* = 0.04)) predicted mortality. A model combining cardiac risk factors including: SBP ≥160 mmHg, DM, obesity and CHD proved the most powerful independent predictor of mortality (OR 3.991 ((1.919 to 6.844); *p* = 0.002) (Table 4).

Mechanical ventilation prediction: CHD (OR 5.321 (1.410 to 9.908)) and obesity (OR 3.872 ((1.771 to 10.72); *p* = 0.02)) predicted the need for mechanical ventilation in a multivariate analysis model. Almost the same model above, combining cardiac risk factors, proved the strongest independent multivariate predictor of the need for mechanical ventilation (OR 1.501 ((0.809 to 6.108); *p* = 0.001)) (Table 4). Collinearity between these measurements was not met based on VIF <10 for all predictors.

## 4. Discussion

Findings: The results of this cohort study reveal the following: (1) Cardiac risk factors were more prevalent among Coptic clergy with arterial hypertension (AH) compared to those without AH; (2) The mortality rate increased linearly with the increasing number of cardiac risk factors; (3) Clergy serving in Northern and Southern Egypt had a higher mortality rate compared to those in Europe and USA combined; (4) The impact of AH on mortality was significant only in Southern Egypt; (5) A model combining cardiac risk factors was the best independent multivariate predictor of COVID-19-related mortality and the need for mechanical ventilation.

Data interpretation: The Framingham study established the important relationship between atherosclerosis risk factors, namely hypertension, diabetes, dyslipidemia, obesity and smoking on cardiovascular disease, particularly coronary artery disease and acute events [10,11]. It has also established the significant benefit of optimum risk factor control on clinical outcome, including survival and acute events, e.g., myocardial infarction and stroke [12,13]. Moreover, at the beginning of 2020, the American College of Cardiology (ACC) proposed clinical guidance focused on the cardiac implication in COVID-19 and recommendations for the Centers for best Disease Control and Prevention [14]. Those recommendations were further strengthened by meta-analyses based on clinical trials, which led to the currently available clinical guidelines of disease prevention [15,16]. Coptic clergy should not be seen as different, based on the abundance of risk factors they carry, as we have previously demonstrated [9]. Obesity was found to be the most prevalent risk factor and the main predictor of the significantly high prevalence of COVID-19 among the Coptic clergy [9,17]. The impact of obesity on the immune system is already well established and explains this high prevalence of COVID-19 among the clergy.

Our current findings go on to highlight the importance of the many cardiac risk factors which may be found among Coptic clergy. This is likely to be related to their lifestyle, particularly the lack of exercise undertaken, as a result of the significantly high demands on their time [18]. However, we are hereby showing clearly that ignoring optimum control of those risk factors, particularly in clergy suffering from COVID-19, puts them at a significantly high risk of mortality and need for mechanical ventilation, with its known problems and uncertain clinical outcome. These findings highlight the serious need for optimum risk factor control, lifestyle improvement and active immune system support. Perhaps a well-designed and balanced education program for Coptic clergy should be devised in different local languages, with recommendations for strict adherence to guidelines. Such a strategy, if seriously and religiously implemented, could only result in improved clinical outcomes.

Clinical implications: Coptic clergy suffering from COVID-19 are at high risk of mortality and need for mechanical ventilation if admitted to intensive care units. This health hazard can be strongly predicted by their cumulative cardiovascular risk factors. Since most risk factors are controllable, a strict education program should assist the clergy in avoiding this risk and in implementing a healthy lifestyle, leading to a stronger immune system.

Severe Covid-19 is often characterized by a dysregulated immune response, leading to lymphopenia and the cytokine storm, which attacks healthy tissue and is often fatal. Excessive cytokine production and their sustained elevation were found to provide a “core” COVID-19 signature; some of these cytokines are associated with blood clotting, another common concern in severe COVID-19. [19] A recent meta-analysis showed that in older patients, hypertension, diabetes and cardiovascular disease, all known inflammatory conditions, conveyed a higher risk of severe COVID-19 and/or mortality, with ORs of 2.5, 2.25 and 3.11, respectively. [20] Obesity, another inflammatory condition, is also a key risk factor for severe COVID-19 and has been shown to weaken the immune system by expanding adipose tissue production of pro-inflammatory cytokines and downregulating anti-inflammatory immune cells such as M2 macrophages, T helper (Th2) cells and regulatory T cells. [21,22] Similarly, in COVID-19 patients with hypertension, pre-activation of the immune cells has been found, manifesting as elevated inflammatory cytokines, which led to an augmented immune response in these patients on contact with the virus and delayed viral clearance [23]. In addition, angiotensin-converting enzyme 2 (ACE2) as aminopeptidase with key role in the cardiovascular and immune systems has been identified as a functional receptor for SARS-CoV2 by binding the spike protein of the virus to ACE2. It is highly expressed in the heart and lungs, particularly in alveolar epithelial cells in patients with COVID-19 and CVD and severe symptoms, in whom it might be associated with increased secretion of ACE2 compared to those without CVD [24].

Study limitations: The analyses undertaken in this study relied purely on the data received from the participants rather than direct control by the investigators. We designed the study and the information spreadsheet, which was sent to all dioceses, with a clear request to complete the data and send it to the principal investigator hence we have no hand in the data completion or accuracy. Not all Coptic dioceses contributed to the study, but there is no reason to suppose that the risk factors were materially different in other dioceses. Our suggestion concerning the expected impact of a specially devised education program remains to be tested and proved.

Conclusions: Coptic clergy carry multiple cardiovascular risk factors, which cumulatively are the most powerful predictors of COVID-19-related mortality and the need for mechanical ventilation.

## Figures and Tables

**Table 1:** Demographic and clinical data between clergy groups.

Variable	Clergy	Clergy AH-	Clergy AH+	*p*
	(*n* = 213)	(*n* = 136)	(*n* = 77)	Value
**Demographic and Clinical Data**				
Age (years)	49.6 ± 12	46.3 ± 11	56.1± 11	<0.001
BMI (m/kg^2^)	31.9 ± 6.2	31.5 ± 6.3	33.1 ± 5.9	0.09
SBP (mmHg)	127 ± 13	121 ± 8.5	135 ± 14	<0.001
DBP (mmHg)	83 ± 9.1	81 ± 6.6	87 ± 11	<0.001
Underweight (n, %)	0 (0)	0 (0)	0 (0)	0.91
Normal weight (n, %)	17 (7.9)	12 (9.3)	5 (6.3)	0.12
Overweight (n, %)	74 (34.7)	54 (39.7)	20 (15.5)	0.02
Obese (n, %)	122 (57.3)	70 (51.7)	52 (68.2)	0.04
DM (n, %)	59 (27.7)	26 (19.1)	33 (43.2)	0.001
Dyslipidemia	68 (31.9)	22 (16.4)	46 (60.6)	0.001
CHD (n, %)	20 (9.4)	10 (7.4)	10 (13.6)	0.04
Family history of CHD (n, %)	22 (10.3)	10 (7.4)	12 (15.6)	0.01
Family history of stroke (n, %)	16 (7.5)	6 (4.76)	10 (10.3)	0.04

AH+, with arterial hypertension; AH-, without arterial hypertension; BMI, body mass index; CHD, coronary heart disease; DM, diabetes mellitus; SBP, systolic blood pressure; DBP, diastolic blood pressure.

**Table 2:** Outcome data between clergy groups.

Variable	Clergy	Clergy AH-	Clergy AH+	*p*
	(*n* = 213)	(*n* = 136)	(*n* = 77)	Value
**Outcome data**				
Home treatment (n, %)	171 (80.2)	110 (81.6)	61 (78.7)	0.55
Hospital treatment (n, %)	36 (16.9)	21 (15.4)	15 (19.7)	0.23
Intensive care (n, %)	17 (7.9)	11 (8.3)	6 (8.3)	0.81
Prevalence (%)	13.6	10.2	20.1	0.001
Mechanical ventilator (n, %)	15 (7.1)	8 (5.9)	7 (9.1)	0.09
Death (n, %)	10 (4.69)	5 (3.68)	5 (6.49)	0.058

AH+, with arterial hypertension; AH-, without arterial hypertension.

**Table 3:** Geographical impact on clinical outcome.

Variable	EU +USA	Northern Egypt	Southern Egypt	*p*
	(*n* = 31)	(*n* = 136)	(*n* = 46)	Value
**Death (n, %)**	0 (0)	7 (5.22)	3 (6.51) ^a,b^	0.001
Clergy AH-	0 (0%)	4 (4.71)	2 (6.21) ^a,b^	0.01
Clergy AH+	0 (0%)	3 (5.88)	1 (7.10) ^a,b^	0.02

^a^*p* < 0.05; Gr. I vs. II ^b^ *p* < 0.05; Gr. I vs. III ^c^ *p* < 0.05; Gr. II vs. III. AH+, with arterial hypertension; AH-, without arterial hypertension.

**Table 4:** Predictors of mortality and mechanical ventilation in COVID-19 patients.

Variable	Univariate Predictors	*p*	Multivariate Predictors	*p*
	OR (95% CI)	Value	OR (95% CI)	Value
**Mortality**				
Diabetes	0.845 (0.045 to 2.896)	0.02	1.003 (0.202 to 3.804)	0.09
Obesity	2.301 (1.002 to 4.094)	0.03	3.403 (1.902 to 4.694)	0.04
AH	0.918 (0.103 to 2.191)	0.04	1.403 (0.802 to 4.001)	0.23
Dyslipidemia	1.031 (0.007 to 4.019)	0.11	2.003 (1.002 to 4.309)	0.33
CHD	1.219 (1.098 to 3.004)	0.001	1.607 (0.982 to 3.051)	0.02
Family history for CHD	0.605 (0.025 to 4.106)	0.21		
Family history for stroke	0.729 (0.171 to 2.649)	0.42		
Diabetes	0.845 (0.045 to 2.896)	0.02	0.146 (0.013 to 1.189)	0.08
Obesity	2.301 (1.002 to 4.094)	0.03	3.174 (0.254 to 9.679)	0.31
AH	0.918 (0.103 to 2.191)	0.04	0.587 (0.003 to 5.191)	0.63
Dyslipidemia	1.031 (0.007 to 4.019)	0.11	0.707 (0.101 to 4.201)	0.63
CHD	1.219 (1.098 to 3.004)	0.001	0.936 (1.082 to 8.517)	0.86
Model *	2.400 (0.509 to 1.400)	0.001	3.991 (1.919 to 6.844)	0.002
**Mechanical ventilation**				
Diabetes	0.641 (0.077 to 3.377)	0.51	0.641 (0.077 to 3.377)	0.63
Obesity	3.872 (1.771 to 10.72)	0.01	3.872 (1.771 to 10.72)	0.01
AH	2.347 (1.197 to 4.501)	0.03	2.347 (1.197 to 4.501)	0.23
Dyslipidemia	1.056 (0.310 to 3.594)	0.87	1.056 (0.310 to 3.594)	0.77
CHD	5.321 (1.410 to 9.908)	0.01	5.321 (1.410 to 9.908)	0.01
Diabetes	0.641 (0.077 to 3.377)	0.51	0.209 (0.027 to 1.616)	0.13
Obesity	3.872 (1.771 to 10.72)	0.01	1.358 (0.273 to 6.748)	0.27
AH	2.347 (1.197 to 4.501)	0.03	0.067 (0.007 to 1.145)	0.06
Dyslipidemia	1.056 (0.310 to 3.594)	0.87	0.098 (0.010 to 7.104)	0.81
CHD	5.321 (1.410 to 9.908)	0.01	3.235 (0.451 to 23.19)	0.24
Model **	1.807 (0.750 to 2.991)	<0.001	1.501 (0.809 to 6.108)	0.001

Model * (SBP ≥ 160 mmHg, DM+, Obesity+, CHD+); Model ** (SBP ≥ 160 mmHg, Obesity+, CHD+); AH, arterial hypertension; BMI, body mass index; CHD, Coronary heart disease; DM, diabetes mellitus; SBP, Systolic blood pressure; DBP, diastolic blood pressure.
